# Theoretical and Practical Leadership in Nursing Curriculum: A Scoping Review of the Literature

**DOI:** 10.1177/23779608251380215

**Published:** 2025-09-25

**Authors:** Dawn Prentice, Farhana Madhani, Jane Moore, Arvith Jhirad, Malak Matus

**Affiliations:** 1 Department of Nursing, 7497 Brock University, St. Catharines, Canada

**Keywords:** scoping review, leadership, nursing, curriculum development, professional competence

## Abstract

**Introduction:**

The purpose of this scoping review was to answer the research questions: What is known from the existing literature about theoretical leadership content offered in the nursing curriculum at the undergraduate or graduate level? and What is known from the existing literature about leadership practicums/experiential learning content in nursing curriculum at the undergraduate or graduate level?

**Method:**

A scoping review using Arksey and O’Malley's Framework (2005) was carried out from February to May 2024. Databases searched included Nursing & Allied Health; PsycINFO; Web of Science; Embase; MEDLINE via Ovid; CINAHL and Education Source via EBSCOhost. Thematic analysis was conducted on the articles that met the inclusion criteria.

**Results:**

Thirteen studies were included in this review. After data analysis was completed three themes were identified: (1) *The What*: What is taught in the theoretical and clinical leadership courses which includes concepts such as interpersonal communication, conflict management, and delegation; (2) *The How*: How theoretical and leadership courses are taught. Within this theme different modalities such as simulation based learning, experiential learning opportunities through clinical practicums, service learning and dedicated educational units were discussed; 3) *The Why*: Outcomes of theoretical and clinical leadership courses, that is improvement in students’ knowledge and leadership skills as well as the development of self-confidence in their leadership skills.

**Conclusion:**

Although there is no overall blueprint listing the leadership concepts and skills that need to be included in nursing curriculum, certain leadership concepts and skills such as interpersonal communication, conflict resolution and delegation are taught as part of leadership curriculum using a variety of educational approaches such as simulation, clinical practicums and classroom learning. However, a standardized nursing leadership curriculum using an evidence-based approach that integrates essential leadership competencies will help nursing students develop confidence, accountability, and readiness as they transition into professional roles.

## Introduction

Leadership is a multi-faceted concept that has been defined in many ways. Merriam-Webster Dictionary (n.d.) defines leadership as “the office or position of a leader: capacity to lead; the act or an instance of leading” (para 1). However, in nursing, leadership extends beyond traditional definitions to incorporate collaboration, advocacy, and evidence-based decision-making that enhance patient care outcomes and improvements in healthcare. For instance, the Registered Nurses Association of Ontario (2013) states leadership “is a relational process in which individuals seek to influence others toward a mutually acceptable goal” (p. 130) and the American Nurses Association (2023) further notes:

A nurse leader is defined by their actions, and not always by a position of authority. Leaders in nursing inspire and influence others to achieve their maximum potential. They use applied leadership in nursing by drawing upon critical thinking skills to manage a team. (para 2)

The term “leadership” can imply a formal hierarchical position in an organization or be informally attained. Nevertheless, strong nurse leaders are necessary in all care setting across the healthcare continuum whether in a formal or informal position. More importantly nursing leadership at the bedside or point of care is necessary for advocating for patients and ensuring quality care. This need for leadership is reflected in the Canadian context, where the Canadian Council of Registered Nurse Regulators [CCRNR] (2019) emphasizes leadership as a key competency for new graduate registered nurses in the 2019 entry-to-practice guidelines.

Similarly, the American Association of Colleges of Nurses note that leadership development is an essential competency (2021).

In some cases, leadership is addressed in terms of the personal attributes of an individual or requisite skills and competencies needed for a leadership position as opposed to a formal role. This demonstrates that the term leadership can be interpreted in different ways which may present a concern when there is an expectation for a newly graduated registered nurse to demonstrate leadership entry to practice competencies. Hence, these definitions collectively emphasize the importance of leadership as a fundamental component of nursing practice.

Given that leadership is an expected entry to practice competency for registered nurses it is not clear what the expectations or learning outcomes the student must achieve and how student nurses learn these competencies. Historically nursing educators have incorporated theoretical leadership concepts and clinical practice applications in their curriculum (Scammell et al., 2020). Yet not every nursing undergraduate curriculum takes the same approach to teaching leadership concepts and may offer a theoretical leadership course or clinical practicum in leadership or both. Alternatively, leadership education in nursing may be woven throughout the curriculum.

As nursing educators, it is important to provide opportunities for students to understand the theoretical components of leadership and facilitate learning opportunities to practice leadership competencies in clinical practicums prior to finishing their nursing program. Yet, what is not clear is what should be included in nursing leadership curriculum. Therefore, the purpose of this scoping review was to systematically examine the existing literature on theoretical and experiential courses in the nursing curriculum at the undergraduate or graduate level to understand how nursing leadership competencies are taught. As Munn et al. (2018) suggest, scoping reviews help identify the extent of research on a topic and its focus, as well as provide an overview of the available evidence and how studies have been conducted. Therefore, by examining the available research, this review aims to highlight gaps, best practices, and emerging trends in nursing leadership education. Findings from this scoping review may inform future curriculum development, ensuring that nursing programs effectively prepare students with the leadership skills necessary for professional practice, and improvement in the healthcare system.

## Methods

### Study Design

A scoping review using the methodological framework outlined by Arksey and O’Malley (2005) was undertaken. This method was chosen as appropriate to summarize and disseminate research findings and identify any gaps in the existing literature (Arksey & O’Malley, 2005, p. 21). Steps of the framework include: (1) identifying research questions; (2) identifying relevant studies; (3) study selection; (4) charting the data; and (5) collating, summarizing and reporting the results (Arksey & O’Malley, 2005). The study followed the PRISMA-ScR checklist.

The research team consisted of three nursing faculty members, a professional librarian and two research assistants (RAs), one who is a graduate student and one a 3^rd^ year undergraduate nursing student. The librarian was involved with the scoping review from the beginning, and assisted with development of the search strategy, provided instruction on the use of the software abstraction system with the students and faculty and provided training in title and data abstraction. The RAs were involved with the title and abstract review as well as the final abstraction of the data. The faculty members (DP, FM, JM) provided oversight of the students for each step of the study.

### Research Questions Identification

The research team proposed the following questions for this scoping review:

1. What is known from the existing literature about theoretical leadership content offered in the nursing curriculum at the undergraduate or graduate level?
2. What is known from the existing literature about leadership practicums/experiential learning content in nursing curriculum at the undergraduate or graduate level?

### Identifying Relevant Studies

#### Search Strategy and Selection Criteria

Databases searched included Nursing & Allied Health; PsycINFO; Web of Science; Embase; MEDLINE via Ovid; CINAHL and Education Source via EBSCOhost using the search terms students, nursing, undergraduate, college, learning, leadership, and curriculum. Studies written in English only, nursing undergraduate or graduate, theory or practicum courses were included in the scoping review. To catch as wide a net as possible no date or formal limits were set. Research team members were encouraged to retain notes, comments, and outlying sources for further review through this research study.

The exclusion criteria included no advanced practice nursing roles, doctoral education, evidence synthesis such as scoping or systematic reviews, and articles with reported outcomes not consistent with the research questions. Advanced practice roles were excluded as the major focus of this scoping review was on undergraduate nursing leadership education preparation to understand what was being taught to nursing graduates who are entering practice. Doctoral education was also not included as this curriculum is focused on an advanced nursing role and not pertinent to entry to practice competencies. The literature search was conducted from February to May 2024. An example of the search strategy can be found in Table 1.

**Table 1.** Example of Search Strategy Using CINAHL Complete via EBSCOhost.

#	Searches	Results
1	(MH "Students, Nursing+")	48895
2	(MM "Students, Undergraduate")	4762
3	(MM "Students, College")	42725
4	1 or 2 or 3	94255
5	(MH "Curriculum+")	49706
6	4 and 5	6516
7	(MM "Leadership")	26332
8	(MH "Learning+")	139961
9	6 and 7	96
10	6 and 8	1590
11	AB (Leadership* and curricul* and nurs*)	608
12	TI (Leadership* and curricul* and nurs*)	16
13	TX leadership* n3 curricul* and TX nurs*	727
14	13 or 12 or 11 or 10 or 9	1485
15	Limit 14 to Language = English and Source Types = Academic Journals	1131

#### Study Selection

The searched articles were uploaded to Covidence screening and data extraction database (2024) for review. The research team met to discuss and confirm the criteria for abstract review. A sample of ten titles were retrieved and each group member independently rated the titles for inclusion and exclusion. The research team met and discussed their reasons for inclusion and exclusion until consensus was reached. The RAs then independently reviewed the abstracts to be included for full-text review. Any abstracts where a conflict arose were reviewed by the first two authors (DP, FM) to break the tie and make the final decision.

**Figure 1.**
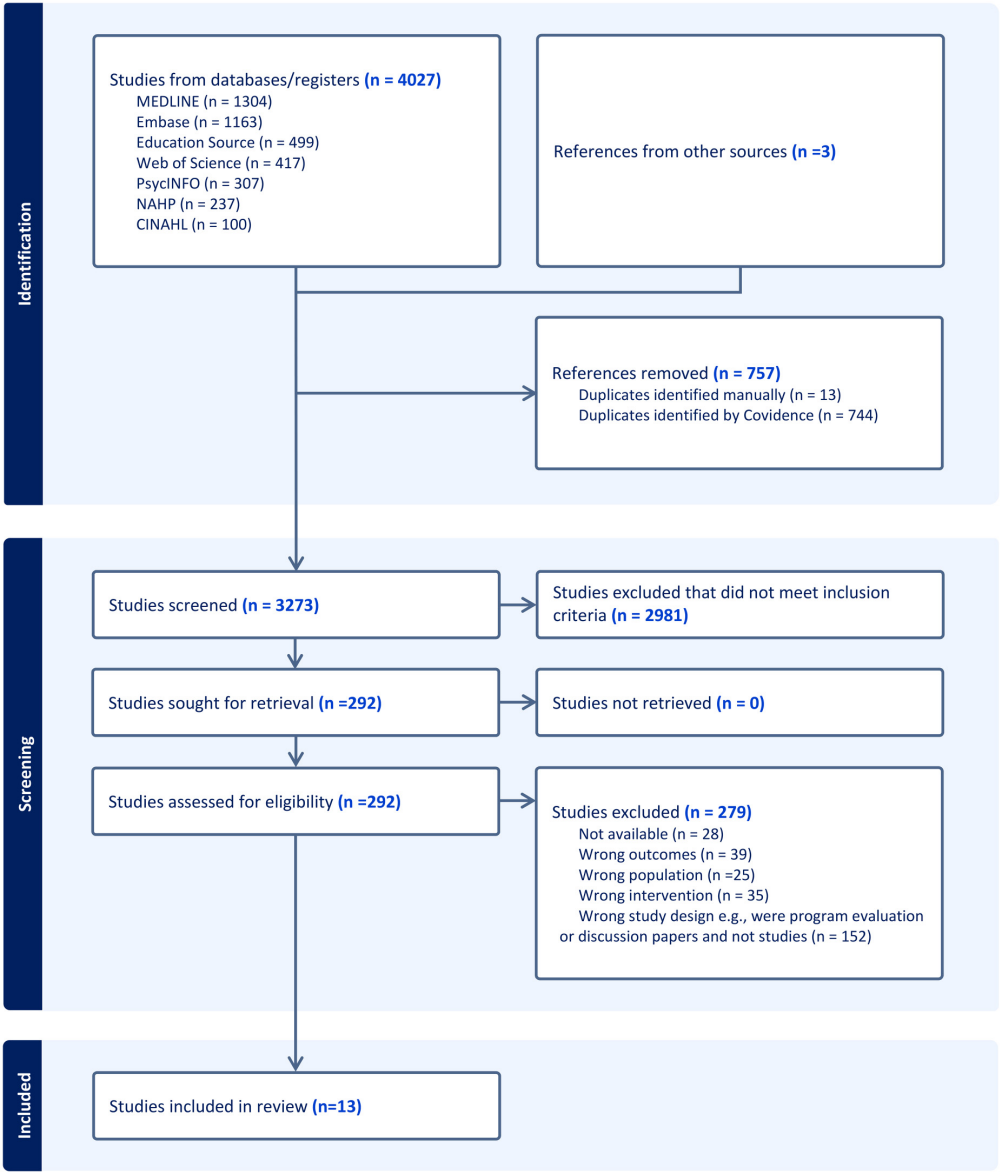
Study Selection Process.

For the second level selection, the articles chosen for full-text review were independently reviewed by each RA and then any conflicts were decided by two faculty members. Because the RAs were students, the faculty members reviewed all the selected full-text articles before final selection. In the final step of study selection, the researchers reviewed alternate or grey literature databases and sources including forward screening and citation references to identify three additional citations for further screening. Hence a total of 13 studies were included for the final review (Figure 1).

PRISMA diagram (Page et al., 2021).

#### Charting the Data

Data from the studies were extracted and presented in an Excel spreadsheet for analysis by the RAs. Information collated on the Excel spreadsheet included: authors and country, study aim, research design, sample, focus on theoretical leadership and/or clinical leadership, undergraduate/graduate, findings and recommendations. The RAs reviewed the final 13 papers and independently uploaded them in separate Excel sheets.

#### Collating and Summarizing of Findings

Data abstracted from the chosen studies were then collated in a separate table for reflexive thematic analysis (Braun & Clarke, 2021). Using an inductive approach two faculty members (DP, FM) independently reviewed the data multiple times before coding and then met to finalize a coding scheme which was then applied to all the data. Inductive reasoning allowed themes and patterns to emerge organically from the data, rather than using a pre-existing framework.

Any discrepancies were discussed until consensus was reached. Three themes were developed from the data analysis and then shared with a third faculty member (JM) who independently reviewed the coding scheme and resulting themes for coherence.

## Results

Thirteen studies comprised of ten articles and three dissertations were included in the final review (Table 2). These studies consisted of four qualitative studies and five pre/post survey designs, three mixed method studies and one descriptive study. Seven studies were authored in the United States, and one from Brazil, Canada, India, Jordan, Saudi Arabia, and Taiwan.

**Table 2.** Studies Included in the Review.

Authors & date & country	Study design	Study population	Interventions	Results
Abou Hasish & Bajbeir (2022) Saudi Arabia	Mixed-methods study	Undergraduate	Clinical sessions focused on management processes, communication, delegation, staffing, quality, and accreditation through hands-on experiences. Simulation sessions used role play to teach leadership styles, problem-solving, job preparation, safety, and incident reporting.	Leadership training and simulation significantly improved students' career planning knowledge and self-efficacy, with gains in managerial, leadership, and career preparation aspects. Positive correlation emerged between career planning knowledge and self-efficacy after the intervention. Students valued debriefs for reflection and appreciated the facilitator's support.
Byrd (2022) United States	Pre-post survey design	Undergraduate	The course covered leadership, communication, conflict resolution, diversity, ethics, and career management through lectures, online videos, and experiential learning. Clinical projects applied leadership concepts in healthcare settings.	The pre-survey showed low leadership knowledge, but active learning improved understanding and confidence across all areas. Significant growth occurred in quality improvement and leadership strategies, demonstrating the course's effectiveness.
Demeh & Rosengren (2015) Jordan	Qualitative descriptive study	Undergraduate	The course ‘Management and Leadership in Nursing-Clinical’ integrates leadership and management concepts into practical healthcare experiences. Fundamental theories and competencies through hands-on activities and nurse leader role discussions, departmental analysis, clinical conferences, evaluations were covered.	Students emphasized the need for role models in nursing and the active practice of clinical leadership, not just observation. They suggested introducing leadership education earlier and recognized its value in bridging theory and practice. Clinical leadership enhanced their organizational, prioritization, and leadership skills, benefiting both their personal and professional lives while improving care quality.
Fetters et al. (2023) United States	Pre-post survey	Undergraduate & accelerated	The research highlights Simulation-Based Learning Experiences (SBLEs) as essential for clinical education, improving knowledge retention, crisis management, and student confidence. The study used a leadership course for senior nursing students, incorporating CBE principles, case studies, role-playing, and SBLEs.	Analysis of pre- and post-education surveys showed significant improvements in 14 of 19 areas, indicating increased self-confidence and communication skills in handoffs during simulation-based learning. Findings suggest competency-based education supports students' transition to clinical practice.
Foli et al. (2014) United States	Pre-post survey	Undergraduate	Service-learning (SL) was a key component of a leadership and management course, integrating peer leadership, community involvement, and social change projects.	Student perceptions of the service-learning project showed a strong correlation with increased understanding of course concepts, except for leadership roles inside or outside the nursing school. A positive association was found between improved delegation skills and other service-learning items like career application, community contribution, and leadership in student organizations. Working with peers on semester-long projects provided a safe environment for students to practice and refine leadership behaviors. Students reported growth in leadership, and their self-assessments aligned with peer observations at the end of the semester.
Galuska (2015) United States	Mixed-methods study	Undergraduate	Throughout the semester, students in the dedicated education unit (DEU) practiced leadership skills under the guidance of the Clinical Instructor (CI), with opportunities such as team collaboration, leading patient care discussions, delegating tasks, and participating in governance meetings. The matching of student and CI schedules for full 12-h shifts provided immersion in the clinical environment. The Clinical Faculty Coordinator (CFC) mentored students and CIs, held post-clinical conferences, and conducted evaluations.	Quantitative results showed significant improvements in self-reported leadership behaviors for students in the DEU, particularly in areas like Model the Way, Inspire a Vision, and Challenge the Process. However, Enable Others to Act showed no significant change. The DEU students' leadership development aligned with the expectations of "apprenticeship learning," where they received hands-on support, practice, and coaching. Mixed methods results highlighted the DEU's positive impact on leadership development, emphasizing the critical role of the CI in guiding students. This supportive relationship helped students build their professional identity and leadership skills.
Ha & Pepin (2018) Canada	Qualitative study	Undergraduate	Clinical nursing leadership, one of the program's competencies, was developed through clinical practice, problem-based learning, and leadership courses. It was assessed formatively during each year and summatively in the third year through two leadership courses. These courses covered topics such as quality improvement, leadership styles, teamwork, change management, and organizational theory, with the educational intervention designed to complement these courses. Videos, journalling and group discussions were utilized.	Videos were highly rated for helping students better understand clinical nursing leadership by providing concrete, relatable demonstrations, which were more effective than reading definitions. Journalling encouraged students to observe clinical leadership, but some found it less useful if they hadn't witnessed leadership in practice. The heavy academic workload made the journal a lower priority for some. Group discussions helped students' understand clinical nursing leadership, though some struggled to connect leadership to nursing or found discussions too theoretical.
Hsieh et al. (2022) Taiwan	Pre-post survey	Undergraduate	Leadership development was supported through lectures, peer activities, and observing teacher behaviors. Leadership competencies were integrated into 12 professional courses using strategies like individual and group projects, clinical experience discussions, critical thinking, and self-reflection.	In Phase 1 of the Delphi process, some experts argued that nursing students wouldn't apply leadership education until clinical practice, suggesting it was unnecessary in undergraduate studies. The NLCAS/UNS was administered before and after the intervention, showing significant improvement in leadership competence on nine items. While a one-year course may be too short for major changes, the study demonstrated that students' leadership competencies improved through a modified curriculum. Students set their own professional goals and visions, which were further developed as they advanced in their education.
Manfredi & Valiga (1990) United States	Descriptive study	Undergraduate & graduate	Data from the National League of Nursing (NLN) Self-Study Reports prepared for accreditation was analyzed for leadership content. A dichotomous scale, developed from a literature review on leadership and management, was used to assess eight components of each report.	Leadership and management were often used interchangeably in nursing courses, with content covering staffing, budgeting, advocacy, and conflict resolution. Only 50% of schools integrated leadership as a curriculum thread, mainly emphasizing advocacy, autonomy, and accountability. Key leadership behaviors like mentoring and power dynamics were rarely addressed, while management activities included role modeling and maintaining stability.
Marath & Ramachandra (2015) India	Quasi-experimental, pre-post-test study	Undergraduate	The leadership development package, based on Kouzes and Posner's five exemplary leadership practices (Model the Way, Inspire a Shared Vision, Challenge the Process, Enable Others to Act, and Encourage the Heart), consisted of four parts and eight modules. It was administered over four weeks, with one day per week, using various teaching techniques including self-assessment, structured learning, skill-building activities, group work, reflective thinking, and ongoing self-learning.	None of the participants had received formal leadership training. It highlighted that primary degrees may not fully prepare nurses for leadership roles, and healthcare organizations need to develop leadership programs to address this gap. The most commonly used leadership practices were "Enable Others to Act," "Encourage the Heart," and "Model the Way," while "Inspire a Shared Vision" and "Challenge the Process" were ranked the lowest. The leadership development package improved all five leadership practices for participants, as reported by both self-assessments and observers.
Miles (2015) United States	Exploratory descriptive study with mixed-methods design	Nurse educators & practice leaders	This section of the survey explored various aspects of the leadership course, including course content (structure, guiding theories, opportunities for leadership skill application, availability of leadership electives, and methods for measuring leadership outcomes. A strong emphasis was placed on identifying how leadership skills and concepts were applied in practice, particularly through clinical practicums and leadership projects. More than half of the participants reported engaging in these opportunities, highlighting their significance in leadership development.	Leadership courses were mostly stand-alone, lacking structured frameworks and integration into the curriculum. Students struggled with leadership concepts, and clinical experiences were seen as essential for leadership development. Gaps in education were noted, particularly in team leadership and unit management. Clinical practicums and interprofessional quality and safety projects were valued. Delegation was frequently mentioned as a key leadership skill, but no standardized framework for leadership competencies existed across programs.
Santos et al. (2021) Brazil	Case study with qualitative approach	Undergraduate	Peter Senge's *The Fifth Discipline* was adopted to reshape nurse-leader training, shifting from a teacher-centered model to one that fosters central learning capacities. Leadership development was supported through simulation labs, workshops, forums, discussions, and practical field activities, all contributing significantly to competency development.	Students recognized that their institution's training model promotes ongoing leadership development, aligning with Senge's principles by fostering aspirations, dialogue, and understanding of complex situations. However, leadership education had gaps in formal teaching despite being embedded in the curriculum. Continuous leadership development throughout the program encouraged teamwork and relational skills essential for care management. Students emphasized the need to integrate leadership training from the start of the program and suggested applying new teaching strategies and practical experiences to enhance learning, reinforcing Senge's focus on reflective dialogue and growth.
Schmidt (2021) United States	Qualitative study with phenomenological approach	Undergraduate	In the Medical-Surgical course, students completed 120 h of clinical practicum over 14 weeks in step-down or progressive care units. Each week, a different student assumed the Team Leader (TL) role, ensuring all students gained leadership experience. TLs oversaw their classmates' responsibilities and documented their experiences in reflective journals.	Participants found managing multiple patients to be the most valuable aspect of the TL role. It helped them develop skills in prioritizing care, organizing medication administration, and ensuring high-quality documentation. TLs also coordinated lunch breaks to maintain organization and support patient safety while ensuring peers received their breaks.

Twelve studies were based on undergraduate nursing programs and one study on undergraduate programs with a small sample of graduate programs. The publication dates ranged from 1990 to 2023. Three of the studies referred specifically to leadership skills developed during clinical practicums (Demeh & Rosengren, 2015; Galuska, 2015; Schmidt, 2021).

Three emergent themes were developed from the data analysis to include: (1) *The What*: What is taught in theoretical and clinical leadership courses; (2) The *How:* How theoretical and clinical leadership courses are taught; (3) *The Why*: Outcomes of theoretical and clinical leadership courses. Each theme will be fully explored in the following paragraphs.

### Theme #1: *The What*: What is Taught in the Theoretical and Clinical Leadership Courses

Included within this theme are the findings reported on leadership components currently being taught. In the studies that examined leadership in clinical settings, observation of clinical leadership and role modeling to improve leadership skills and observe leadership skills being demonstrated daily in work settings, were examples of what is being taught. Providing the opportunity to incorporate leadership theory into the practice setting was a method used to address the theory to practice gap. Notably students in one study reported that clinical leadership needs to be practiced in the clinical setting (Demeh & Rosengren, 2015).

Interpersonal communication, an important aspect of leadership skills, was mentioned in many of the studies focusing on both the theoretical and practical content of leadership development (Abou Hashish & Bajbeir, 2022; Byrd, 2022; Demeh & Rosengren, 2015; Schmidt, 2021). Learning about communication channels using a case study approach (Abou Hashish & Bajbeir, 2022) or in a simulation based learning opportunity as part of a team leader experience (Fetters et al., 2023) are examples of the leadership content in the curriculum.

Acquiring knowledge about delegation and how to delegate to others was another leadership competency addressed in this theme (Abou Hashish & Bajbeir, 2022; Byrd, 2022; Demeh & Rosengren, 2015; Schmidt, 2021). Delegation is one leadership competency that nursing students do not often have the opportunity to practice while in a student role. Miles (2015) reported that delegation was frequently mentioned as a leadership skill students were lacking.

Examining quality improvement (QI) principles and impact on patient safety were other leadership concepts reported in the studies (Abou Hashish & Bajbeir, 2022; Byrd, 2022; Fetters et al., 2023). These studies discussed student knowledge and comfort with topics such as QI principles and processes including root cause analysis, patient safety principles, and change management. Having students understand the link between patient safety principles and prevention of clinical incidents is a valuable leadership skill.

An older study by Manfredi and Valiga (1990) discussed content that was included in leadership/management courses including staffing ratios, staffing assignment, budgeting, and patient advocacy. Interestingly at that time they reported that 50% of the nursing schools addressed leadership as a thread in the curriculum. In her dissertation, Miles (2015) reported that “although certain skills were identified repeatedly there was no commonly accepted framework outlining leadership competencies to be addressed in all pre-licensure baccalaureate programs” (p. 95–96).

Byrd (2022) outlined other leadership topics such as teaching about effective leadership strategies, the role of the nurse and discussion of politics. A focus on career planning as part of leadership content was mentioned by Abou Hashish and Bajbeir (2022).

### Theme#2: *The How*: How Theoretical and Clinical Leadership Courses are Taught

This theme encompasses how leadership components in theoretical and clinical courses are taught. Many of the studies reviewed involved a type of experiential learning activity. One such pedagogy simulation-based learning, was used to advance knowledge about leadership competencies was discussed in two studies (Abou Hashish & Bajbeir, 2022; Fetters et al., 2023). Abou Hashish and Bajbeir (2022) focused on theoretical leadership content and discussed using simulation to teach management and leadership concepts and career planning. The authors emphasized that a debriefing session post simulation is crucial for students to reflect on their actions and identify areas for their improvement. Fetters et al. (2023) also used a simulation-based approach to provide students with the opportunity to serve as a team leader and conduct a simulated hand-off during a patient emergent event as a method of teaching a leadership competency.

Demeh and Rosengren (2015) reported on a qualitative study of senior level clinical student experience where students were able to see clinical leadership in real time through the role modeling of clinical supervisors. Moreover, another study examined students’ experiences of assuming a team leader role during clinical practicum and how this “hands-on learning” of leadership skills and team leader experience enhanced students’ knowledge and skills (Schmidt, 2021).

In a Dedicated Education Unit (DEU), another type of experiential learning setting, students during their practicum on a hospital-based unit were able to see leadership attributes being modeled with the clinical instructor also being an effective role model for student development of clinical and leadership skills (Galuska, 2015). Service learning was another method of experiential learning used for teaching leadership in addition to the traditional classroom setting. Through planning an end of term health fair students had the opportunity to apply leadership skills (Foli et al., 2014). The semester-long service-learning project was in addition to weekly lectures on management and leadership constructs.

One study addressed teaching clinical leadership skills in first year nursing students as opposed to senior level nursing students. Using videos and having students observe these nursing role models and journal their impression about the videos as well as having discussions with students about nursing leadership, the authors used a multi-modal approach to teaching leadership qualities (Ha & Pepin, 2018). Other teaching strategies used to teach leadership skills included scheduled workshops (Marath & Ramachandra, 2015; Santos et al., 2021) and use of active learning strategies which includes lectures, virtual instruction, and online supplementary reading materials (Byrd, 2022).

### Theme #3: *The Why*: Outcomes of Theoretical and Clinical Leadership Courses

This theme addresses the leadership outcomes or competencies noted in the reviewed studies. Improvement in students’ knowledge about managerial and leadership skills were outcomes discussed as well as the development of self-efficacy (Abou Hashish & Bajbeir, 2022). Career preparation and self-directed goal setting were also identified as outcomes (Abou Hashish & Bajbeir, 2022; Hsieh et al., 2022). The opportunity to incorporate leadership skills in their practicum and further develop leadership competency were identified learning outcomes (Galuska, 2015; Hsieh et al., 2022). Improvement in student self-confidence and comfort communicating information was another outcome identified, using a simulated patient hand-off experience (Fetters et al., 2023).

In studies that discussed clinical leadership during a practicum, the authors reported that through these leadership practicums patient safety is promoted, and students have an increased awareness of organizational and leadership skills. Clinical practicums also provide students with the opportunity to practice these skills prior to graduation and develop leadership competencies (Demeh & Rosengren, 2015; Schmidt, 2021).

Growth in leadership skills by evaluation of others in a service-learning project (Foli et al., 2014) was identified as a learning outcome and learning to understand the difference between “leadership and management” (Manfredi & Valiga, 1990; Miles 2015) were also noted as outcomes.

The answer to the two research questions were interwoven in the three themes and cannot be easily separated as all the themes had both a theoretical and experiential component. For example, effective communication, an essential leadership skill, can be taught from a theoretical perspective but also applied and refined in an experiential setting such as a clinical simulation or during a clinical practicum. Another example, learning about delegation where the rules or parameters around delegation can be taught in a classroom but the experiential application in a simulation exercise or during a clinical practicum can further highlight development of this skill and provide the student with the confidence to achieve this skill. In the third theme, which addresses outcomes, a theoretical component that is knowledge of the concept being taught as well as an experiential component such as a clinical practicum, or simulation is required for the student to develop confidence in achievement of the skill.

## Discussion

The aim of this scoping review was to examine the existing literature on theoretical and experiential courses in the nursing curriculum at the undergraduate level or graduate level to understand how nursing leadership competencies are taught. The research team found that there is no overall blueprint or standardized curriculum for what needs to be included in leadership curriculum. This is important as concepts of leadership are included in the National Council of State Boards of Nursing (2023) NCLEX-RN exam, under the management of care section, and includes elements such as client advocacy, prioritization of care, collaboration with other healthcare providers, delegation and participation in QI activities (p. 8). New nursing graduates must have knowledge of these elements for preparation for their exam and as noted earlier leadership competencies are an expected entry to practice competency for new graduates. Therefore, nursing graduates must have knowledge and skills in these areas and a standardized leadership curriculum would be helpful to ensure all nurses are prepared with leadership skills.

One of the main findings was the different modalities for teaching leadership skills or competencies, including simulation (Abou Hashish & Bajbeir, 2022; Fetters et al., 2023), journaling (Ha & Pepin, 2018), as well as experiential opportunities in clinical settings (Demeh & Rosengren 2015; Galuska, 2015), where students can practice leadership skills such as delegation and communication of patient hand-offs in real time. All these teaching approaches provide students with the opportunity to self-reflect on their leadership skills which raises students’ awareness of the required leadership skills as well as assists students with identification of leadership skills for further growth. The literature focusing on simulation in nursing education reveals that it provides opportunity for students to enhance their clinical skills, critical thinking, and decision-making in a safe, controlled environment (Welch et al., 2019).

An integrative review of 10 articles suggests that incorporating simulation into nursing management and leadership courses can enhance students’ skills in delegation, problem-solving, decision-making, communication, and teamwork. The author recommends incorporating simulations into undergraduate nursing leadership courses (Labrague, 2021).

Simulation-based learning also enables students to apply their learned skills when practicing in real-life scenarios, which would improve their confidence and patient care outcomes. The recent publication by Dopelt et al. (2023) affirms that simulation offers benefits beyond traditional face-to-face learning by providing practical experience and strengthening various leadership abilities.

Valdes and colleagues (2021) in particular, discuss the effectiveness of an escape room simulation to improve nursing teamwork, leadership, and communication skills using a pre- and post-design method. The authors revealed that an escape room can impart experiential learning and critical skills needed to work as an effective member of a team.

Similarly, there is a wide range of literature focusing on the importance of journaling or reflective practice in nursing education. Journaling is a valuable tool for enhancing critical thinking, self-awareness, and professional growth. It allows students to analyze their experiences, identify strengths and areas for improvement, and develop problem-solving skills (Hwang et al., 2018; Miraglia & Asselin, 2015).

Three studies discussed an important aspect of leadership and that is promoting patient safety (Byrd 2022; Demeh & Rosengren, 2015; Schmidt 2021). Safety is cornerstone to quality patient care, and the research team suggest leadership content and skills may be made more understandable to undergraduate nursing students when taught in relation to patient safety and quality of care. Using patient safety as a foundation for teaching leadership skills may be worthy of further investigation.

'Soft skills” is a common term heard today. Cimatti (2016) notes that soft skills encompass language and communication abilities, friendliness and the ability to work in a team. Laari et al. (2024) offered a concept analysis of soft skills in nursing which include the “intrapersonal traits, interpersonal skills, and the creativity of the nurse that through professionalism, teamwork and good communication skills leads to quality of nursing care and improvement of patient/client outcome and satisfaction” (p. 247–248). The research team suspects that leadership skills considered to be “soft skills” such as effective communication skills, conflict resolution, and being a team player, are currently embedded in many nursing curriculums and may be couched as professionalism in nursing courses or as threads in the curriculum.

In the early stages of this review, it became evident that leadership was mentioned as a key word in many studies, yet the term was used in very expansive and vague manner. Another finding was that many of the abstracts reviewed were studies that focused on different teaching pedagogies as opposed to curriculum on leadership. Additionally, there were few studies that addressed the leadership outcomes or desired competencies and were more focused on evaluation of teaching approaches.

Also, the research team noted that most of the literature focusing on leadership skills discuss the key required skills for acute care settings, which is the traditional focus of nurses' roles. Future areas to explore: Will there be variability in the curriculum competencies for nurses who aim to practice in the community or long-term care? and what priority leadership competencies should students be learning to prepare them for their role?

### Strengths and Limitations

One of the strengths of this review is that, even though it includes a limited number of studies, it highlights where evidence is lacking and provides a strong rationale for further research. The review also identifies the need to develop a blueprint or framework for leadership curriculum in nursing. This is especially important as nursing is no longer limited to bedside care; nurses must now be prepared to take on broader roles in today's changing healthcare environment.

The research team included studies only written in English which may have limited studies that addressed the research questions. The research team members used unique comprehensive search strategies for each scholarly database. These search strategies and noted inclusions and exclusion screening criteria may have missed relevant studies due to the inherent challenges of searching. Half of the studies were authored in the United States, and this may limit the applicability of findings to diverse global nursing especially in areas where registered nurses may not be viewed as leaders in health care. Additionally, this review was not able to capture the long-term impacts of leadership education on nursing graduates’ leadership roles, career advancements, or contributions to healthcare, therefore future reviews should focus on these parameters.

### Implications for Practice

Developing an effective nursing leadership curriculum has more significant implications for nursing practice, patient outcomes, and evolving healthcare systems than ever before.

It is important that nursing educators equip their nursing students with strong leadership skills so that nursing graduates and future nurses can effectively navigate the changing healthcare system, overcome daily challenges, work collaboratively with interdisciplinary teams, and, most importantly, advocate for their patients.

In the absence of blueprint or a standardized curriculum framework and variability in the outcome measures, this review suggests (a) that future studies should focus on articulating the specific leadership competencies that are most critical for undergraduate nursing students and (b) identify effective teaching approaches to address these competencies; (c) examine the long-term career outcomes for nurses who receive structured leadership training during their education and how they aligned with the evolving health care needs and (d) conduct a survey of nursing schools to determine what is currently included in their leadership curriculum.

## Conclusions

Much of the literature reviewed in this scoping review focused on the how leadership skills within baccalaureate nursing programs are taught, and the innovative teaching strategies, and practical applications. The key focus of the nursing leadership curriculum requires a comprehensive and evidence-based approach that integrates essential competencies and how these competencies foster confidence, accountability, and readiness among students as they transition into professional roles. The role of the nursing educators is to continuously assess and update course content to align with emerging healthcare trends, leadership expectations, and interdisciplinary collaboration.
